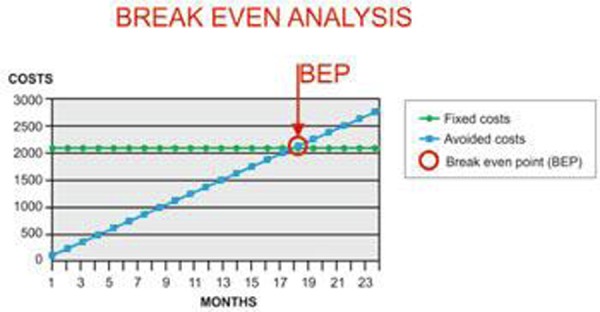# Economic Evaluation of “Pulse Dose” Radiofrequency in the Treatment of Occipital Neuralgia Headache

**Published:** 2012-04-30

**Authors:** Vittoria Giovannini, Rachele Pusateri, Viera Russo, Daniela Viscardi, Rosa Palomba

**Affiliations:** 1 Department of Biomedical, Electronic and Telecommunications Engineering; 2 Department of Surgery, Anesthesiology and Emergency-Resuscitation “Giuseppe Zannini” University “ Federico II” Naples- Italy (giovannini47@libero.it)

**Keywords:** Headache, Radiofrequency, Occipital Neuralgia

## Abstract

Headache occipital neuralgia is an example of pain-disease for which treatment both pharmacological protocols and invasive methods are used. Among the latter, the RF (Radiofrequency) pulse-dose has been of interest for the prospects of analgesic efficacy, safety and patient compliance, although at the moment only data concerning the pulsed RF and not the RF pulse-dose, that represents its evolution, are discussed in scientific literature. The purpose of this study is a “simple” economic evaluation of this method in headache occipital neuralgia.

## I. INTRODUCTION

Essential headache recognizes a significant economic impact, the assessment of which of course are not only included the costs of treatment. There is no single therapeutic option and, if on the one hand the drug therapy is to be distinguished in therapy of acute and prophylactic therapy, the other for the latter no protocol showed a clear superiority over other (1-2-3). This is particularly interesting considering that the same Guidelines issued by scientific societies (4) are evolving as regards both the acute and prophylactic therapy, and that, where indicated and possible, is expected to draw even invasive methods instead of medications (5–6). These procedures must comply with safety rules and still have accost to be taken into account to assess not only their effectiveness, but also the real efficiency (7). Pulse-dose Radiofrequency represents the technological evolution of Pulsed Radiofrequency. It allows to provide pulses or “Doses” with defined and accurate current or voltage values. In fact, in Pulse Dose pulses are maintained constant over time at 45 volts, of 20 ms duration and a temperature not exceeding 42 ° C, compared to pulsed radio frequency in which the pulse amplitude is 45 volts and the maximum temperature of 42 ° C, but if it exceeds 42 ° C, the successive pulses will have a potential of less than 45 volts. On the contrary, in the Pulse Dose, the temperature is kept constant thanks to the infusion of saline solution near the tissue subjected to the procedure. This allows furthermore to shorten procedure time and sometimes to avoid the use of the surgery room. In Pulse Dose radiofrequency pulses are defined previously by the user either as applicable Volt to the electrode, either as applied temperature and as number of doses ranging from a minimum of 120 to a maximum of 2400. In this way doses are provided constant in both voltage value and temperature and the final result is represented by an increase of 70% effectiveness application. Just the ability to set the number of “doses” to be provided is the advantage of this method which can thus be standardized for each peripheral nerve to be treated. Its ideal applications are on peripheral nerve fibers, such as: Great Occipital, suprascapular; Pudendal, Facet Syndrome, Carpal Tunnel. The recent introduction into clinical practice of this method refers to the need to evaluate the analgesic efficacy and possible economic advantage, also in consideration of the fact that, being neuro-modulatory, the RF pulse dose is not a procedure “final”, but presumably its effects will have a limited lifespan and this may affect the cost-benefits ratio.

## II. METHODOLOGY

The aim of our study was to verify the presence of any economic advantage for the Health System of the Campania region resulting from the RF pulse dose compared to headaches drug protocols. In our economic analysis we have used the break even analysis, which identifies the point (= break even point), in which the revenues obtained (avoided costs) equal the total costs incurred for a period of 24 months. We considered as avoided costs the prices of drugs administered to a group of 22 patients (12 M -10 F, age 27–52aa, not suffering from liver failure, kidney or heart failure), afferent (from January to September 2011) to Pain clinic of the University “Federico II” of Naples, suffering from occipital neuralgia and subjected first to drug therapy and, in a second time, the pulse dose RF. The total costs, although generally comprising a fixed part and a variable part, in our case, detailing the costs of treatment with Pulse Dose RF, are only fixed. (ticket for diagnostic anesthetic block to be performed before the RF, DRG for the procedure). Regarding drug therapy, we considered the monthly cost per patient, the weighted average based on three different protocols followed by our patients (and validated in the literature). Cost are reported by (8) 15 patients were taking topiramate 100mg/die, propranolol CR 80mg/die, dexamethasone 4mg/die, rabeprazol 20 mg / die; 4 patients receiving topiramate, dexamethasone, and rabeprazole in dosages above and flunarazin 10 mg / die instead of the beta-blocker; 3 patients taking lamotrigine 100mg/die, pregabalin 300mg/die in addition to the steroid and the inhibitor of the pump. These 22 patients asked, at a later time, an alternative to pharmacological therapy, for these reasons: 10 felt they did not obtain the desired relief, 5 no longer wanted to take “many” drugs, 7 had achieved a fair level of analgesia but at the cost of increased side effects. These patients were then subjected to a session of Pulse Dose RF, providing pulses (“doses”) effective and stable in time and amplitude of 45 V and a duration of 20 ms, for a total of 1200 doses. The infiltration points were chosen based on anatomical descriptions of Vitale and coll., Becser and coll., Loucas and coll., Natsis and coll. (9-10-11-12).

## III. RESULTS

At the end of our economic evaluation, the fixed costs amount to 2,115 euros, while the avoided costs for 12 months amounted to Euro 1416, where the break even point is set at 18 months (Fig. 1).

In practice this means that the Pulse Dose RF would be economically advantageous for the Campania Region SS with respect to drugs only if the term had analgesic efficacy equal to or greater than 18 months.

In our patients the Pulse Dose RF resulted in a reduction of VAS greater than 60% compared to baseline with a significant improvement in quality of life within three and six months from performing the procedure.

## IV. DISCUSSION

From a strictly economic point of view, our study presents a simple model of cost-effectiveness and indicates that in practice if you want to conduct efficiency studies of the Pulse Dose RF, you should perform a follow-up of at least 18 months. On the other hand, for the specialist in pain management, the minimum time limit of 18 months has a relative weight if it occurred to her patients a significant reduction in pain with improved quality of life and overall satisfaction for a significant shorter period. In fact, considering the socio-economic impact of a disease and its treatment, medication-economic studies should take into account several aspects: Van Zundert (13), in an article in 2005, brought this action to a specific Medical Technology Assessment (MTA), which can determine both the total importance (“weight”) of the disease (for the patient and the society) and the overall cost of the treatments, also evaluating their efficacy.

In describing this method, the Author follows the pattern presented previously by Tugwell and coll.(14) that considers the cost of the disease in terms linked to society and quality of life of patients and in terms of costs of attaining a correct diagnosis (laboratory and / or radiological tests) but also the effectiveness of treatments in terms of therapeutic effect, according to guidelines already drawn up or being implemented, and cost-benefit (15). It must also take into account in assessing the costs of chronic disease should be included:

- Direct costs, specifically attributable to its diagnosis and/or therapy (instrumental and laboratory tests, prices of drugs and invasive procedures).
- Indirect costs, which relate to the psychological and social scope of the patient (quality of life, work and recreational activities), and the impact caused to the company (decreased job activity).

It ’s clear that the former are easily quantifiable, while the latter can be investigated only through the administration of particular questionnaires that assume a statistically significant value only on large patient populations, in the condition of homogeneity. Improving the quality of life and patient satisfaction ultimately represents a reduction of indirect costs and, if you had the opportunity to monetize QoLi score improvement, this would affect positively on the economic advantage. Currently, the tests used to measure various aspects of quality of life are subjective and, to have statistical significance, requires a large and homogeneous sample.

## V. CONCLUSION

Currently, this work aims only propose some reflections on a promising technique used for occipital headache as other painful conditions, but for which the scientific literature needs better and more systematic studies. Finally, considering the limited data in the literature, we could evaluate the efficacy and efficiency of the pulse dose RF and continuous RF in occipital headache. The pulse dose RF is a technique of neuromodulation, so that its effect is reversible and repeatable. Giving a response limited in time, however, it will be necessary for the patient to undergo several sessions after months (the duration of effect is subjective). The continue RF, instead, is a neurolesive technique: it offers the advantage of a long-lasting analgesic effect but also the risk of developing deafferentation pain.

**Figure:**